# Investigating the Relationship between Timing of Tweets and Mental Health, Well-being and Sleep Quality in a UK birth cohort

**DOI:** 10.23889/ijpds.v8i3.2284

**Published:** 2023-09-11

**Authors:** Daniel Joinson, Oliver Davis, Nina Di Cara, Nello Cristianini, Claire Haworth

**Affiliations:** 1 School of Electrical, Electronic and Mechanical Engineering, University of Bristol; 2 The Alan Turing Institute, MRC Integrative Epidemiology Unit at the University of Bristol, Bristol Medical School; 3 School of Psychological Science, University of Bristol; 4 Department of Computer Science, University of Bath; 5 School of Psychological Science (University of Bristol), The Alan Turing Institute

## Introduction & Background

Social media use has been proposed as a cause of worsening mental health and wellbeing over the last decade, but its role in mitigating some of the effects of social distancing during the pandemic showed that it also has the potential to improve these outcomes. Whilst existing research disagrees on the degree to which social media use harms or helps, there is growing consensus around the need to move from global measures of social media use to specific measures of types of social media use. These new measures can enable an exploration of proposed mechanisms and causal pathways linking social media use and mental health and wellbeing. A commonly proposed mechanism is nighttime social media use reducing sleep quality, and consequently harming mental health and wellbeing.

## Objectives & Approach

We aimed to investigate the relationships between the time Twitter users post content and their mental health, wellbeing and sleep quality using direct measurements of Twitter use linked to standardised mental health measures in a well-characterized cohort.

This study uses approximately 1.5 million Tweets harvested between January 2008 and March 2023 from 622 participants in the Avon Longitudinal Study of Parents and Children (ALSPAC). These Tweets have been linked to questionnaire data collected on six occasions spanning April 2019 to May 2021. These questionnaires included standard measures of depressive symptoms, anxiety symptoms, mental wellbeing and difficulty sleeping.

We have taken two approaches to explore these relationships, using circular statistical methods novel to social media data analysis to account for day/night cycles. The first approach used mixed effect models to investigate the association between the time a Tweet was posted and the mental health, mental wellbeing and sleep quality of the poster. The second approach explored the relationships between the mean hour participants post Tweets in a given time period, and their mental health, mental wellbeing and sleep quality.

## Relevance to Digital Footprints

This research is highly relevant to Digital Footprints, due to its use of data directly extracted from a social media site. The methodologies employed in analysing this alongside more traditional epidemiological survey data provides an example of how digital footprint data can complemented by high quality ground truths.

## Results

There was evidence that the timing of Twitter activity was predictive of the mental wellbeing and sleep quality of participants, even after adjustment for demographic, educational and socio-economic covariates. However, the hour a Tweet was posted at explained very little of the variation in the mental wellbeing or sleep quality of the participant who posted it (0.1% and less than 0.1% respectively). There was weak to no evidence that the timing of Twitter activity was predictive of the depressive and anxiety symptoms of participants.

## Conclusions & Implications

Whilst this study found evidence that the hour participants post on Twitter is predictive of their mental wellbeing and sleep quality, the amount of variation explained by these models suggests that this is not a clinically relevant risk factor. This study supports arguments in the literature that the use of social media has a very small and insignificant effect on mental health, wellbeing and sleep quality.

